# Diagnostic safety and quality optimization in sepsis study protocol

**DOI:** 10.1002/jhm.70052

**Published:** 2025-04-13

**Authors:** Sachita Shrestha, Marc Kowalkowski, Sarah Birken, Jessica Palakshappa, Jessie King, Chadwick Miller, Jason Pogue, Stephanie Taylor

**Affiliations:** ^1^ Department of Internal Medicine, Division of Hospital Medicine University of Michigan Ann Arbor Michigan USA; ^2^ Wake Forest University School of Medicine Winston‐Salem North Carolina USA; ^3^ Department of Clinical Pharmacy University of Michigan College of Pharmacy Ann Arbor Michigan USA; ^4^ Institute for Healthcare Policy and Innovation University of Michigan Ann Arbor Michigan USA

## Abstract

**Background:**

Sepsis ranks among the “Big Three" conditions most prone to harmful diagnostic errors. Despite its high prevalence and severity, health systems lack effective and contextually tailored strategies to optimize diagnostic accuracy for sepsis.

**Objectives:**

The purpose of this study is to understand factors related to high sepsis diagnostic accuracy using principles and tools of safety and implementation science.

**Methods:**

This is a multi‐site study involving 20 hospitals across four states in the United States. The primary objectives are to (1) describe hospital‐level variability and understand barriers and facilitators to sepsis diagnostic accuracy and (2) apply cross‐case and coincidence analysis to determine minimally sufficient and necessary conditions for optimal sepsis diagnosis that minimizes under‐ and overtreatment. To identify barriers and facilitators of acute sepsis diagnosis, we will conduct electronic surveys and in‐depth interviews with key informants from each hospital. We will use data from electronic health records (EHR) and data warehouses to operationalize sepsis diagnostic accuracy.

**Results:**

We have enrolled 20 hospitals and begum data collection. The findings of this study will be used to develop a context‐specific toolkit that guides the selection of feasible and important strategies to promote optimal sepsis diagnosis in diverse hospitals settings.

**Conclusions:**

The study uses tools and principles from safety and implementation science to generate first‐of‐its‐kind evidence to improve diagnostic excellence in sepsis.

## INTRODUCTION

Sepsis—life‐threatening organ dysfunction due to infection—results in 19 million hospitalizations and over 5 million deaths each year, rendering it the costliest condition and most common cause of in‐hospital mortality in the United States.^1^, ^2^, ^3^, ^4^, ^5^, ^6^ Prompt antibiotic delivery reduces mortality among patients ultimately diagnosed with sepsis.^7^, ^8^ However, establishing a timely and accurate sepsis diagnosis is difficult because of its nonspecific and variable presenting signs and symptoms—approximately one in two patients with suspected sepsis are misdiagnosed.^9^ In the absence of a definitive diagnostic test for sepsis, hospitals have implemented interventions to improve sepsis diagnosis, such as electronic detection alerts, bundles, guidelines, and incentives. Yet, these investments have not consistently improved patient outcomes,^10^, ^11^, ^12^, ^13^, ^14^, ^15^, ^16^, ^17^, ^18^ and sepsis diagnostic errors remain a major quality gap.

Existing research on sepsis diagnosis interventions is lacking in three key areas limiting hospitals’ ability to achieve sepsis diagnostic excellence: (i) Empiric evaluations of sepsis diagnosis interventions focus on avoiding sepsis undertreatment with little attention to sepsis overtreatment, (ii) Although sepsis diagnosis occurs in complex organizational environments, existing research neglects evaluation of key contextual factors that impact sepsis diagnostic accuracy, and (iii) Hospitals lack guidance for early sepsis management that tailor effective sepsis diagnosis interventions to local contextual conditions in which they are likely to be effective.

**Figure 1:**
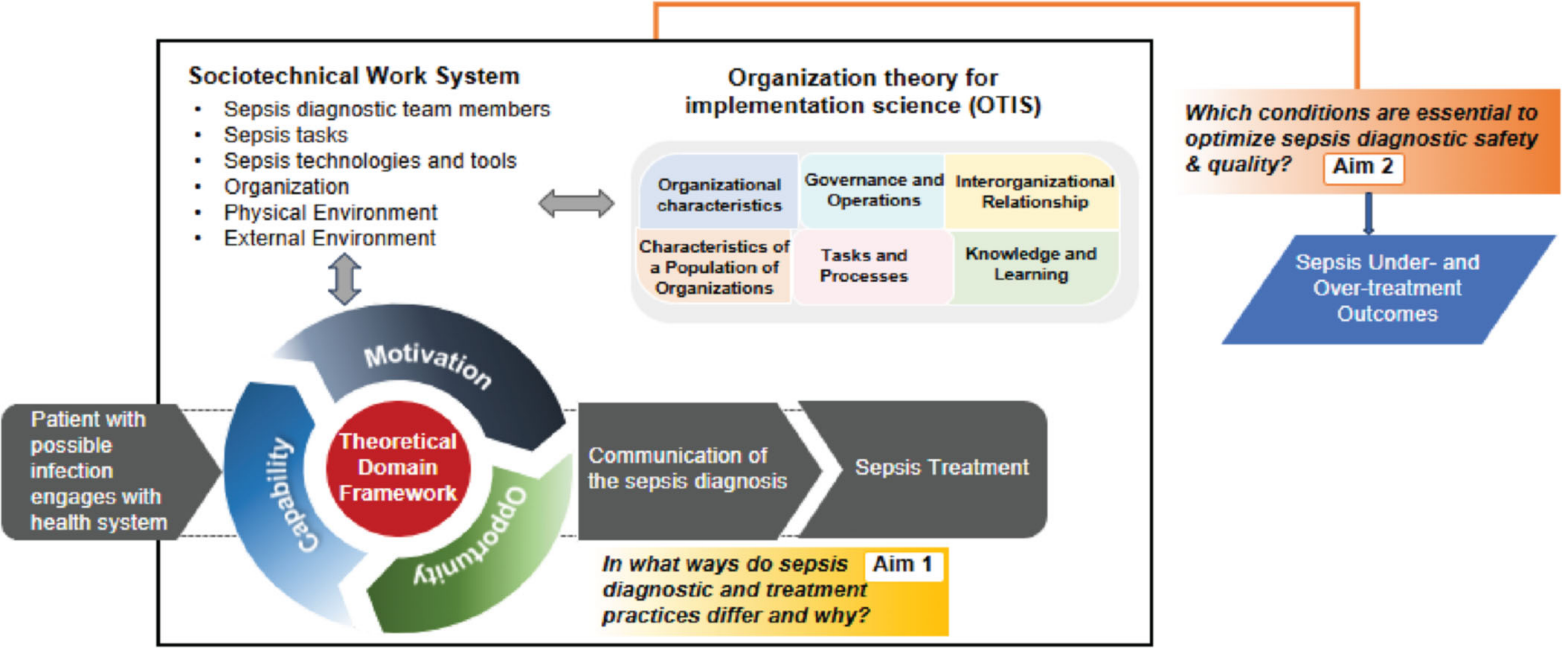
Conceptual model.

The goal of this study is to improve sepsis diagnostic accuracy by applying principles from safety and implementation science. Specifically, the study aims to: (1) examine hospital‐level variability in sepsis diagnostic practices and identify barriers and facilitators that influence accurate diagnosis, including their impact on both undertreatment and overtreatment of sepsis, and (2) determine the specific combinations of diagnostic practices and contextual factors that are minimally sufficient and necessary for achieving high performance in sepsis diagnosis while balancing the risks of under‐ and overtreatment (see Figure 1: conceptual model).

## METHODS AND ANALYSIS

### Study setting

We will include 20 hospitals from two healthcare systems (Michigan Medicine and Atrium Health/Wake Forest) across four states (Atlanta, Georgia, Michigan, and North Carolina) purposively selected to maximize diversity of location, system structures, and patient populations (see Table 1: performance sites). The diversity of clinical care, common electronic health record (EHR) platforms, and large patient volumes make the study sites an ideal environment to evaluate variation in factors affecting sepsis diagnosis. We will begin collecting survey and interview data as well as integrating EHR data from different hospitals starting March 2025.

**Table 1:** Name and locations of performance sites.

S.N	Performance sites
Atrium Health (AH)	
1	AH Cabarrus, North Carolina
2	AH Carolina Medical Center, North Carolina
3	AH Cleveland, North Carolina
4	AH Kings Mountain, North Carolina
5	AH Lincoln, North Carolina
6	AH Mercy, North Carolina
7	AH Pineville, North Carolina
8	AH Stanly, North Carolina
9	AH Union, North Carolina
10	AH University City, North Carolina
11	Wake Forest (WF) Baptist, North Carolina
12	WF Davie, North Carolina
13	WF High Point, North Carolina
14	WF Lexington, North Carolina
15	WF Wilkes, North Carolina
16	AH Navicent, Georgia
17	AH Navicent Baldwin, Georgia
18	AH Floyd Medical Center, Georgia
19	AH Floyd Cherokee, Alabama
University of Michigan	
**20**	Michigan Medicine, Michigan

### Participant eligibility criteria

#### Emergency department key informants

We will include the following organizational roles relevant to sepsis diagnosis: nurses, physicians, and pharmacists practicing Emergency Medicine, Hospital Medicine, Critical Care, and Infectious Disease, organizational quality leaders, sepsis initiative leaders, and antimicrobial stewardship teams.

#### Patients

Patients are eligible if they meet the following criteria: (1) 18 years or older at the time of admission, (2) hospitalized through the emergency department (ED) admission, and (3) presented with possible infection between July 2022 and June 2024, defined as meeting 2 or more Systemic Inflammatory Response Syndrome (SIRS) criteria within 6 h of ED arrival (i.e., temperature >38°C or < 36°C; heart rate > 90 beats/minute; respiratory rate > 20 breaths/min; white blood cell count > 12,000/mm³ or < 4000/mm³, or > 10% bands). SIRS criteria are: (i) consistent with host response to infection and (ii) routinely used to support the diagnosis of infection in practice. We will exclude patients who are transferred from another acute care facility, discharged within 6 h of ED arrival, and have directives for Comfort Care or Palliative Care.

#### Data collection

**Figure 2:**
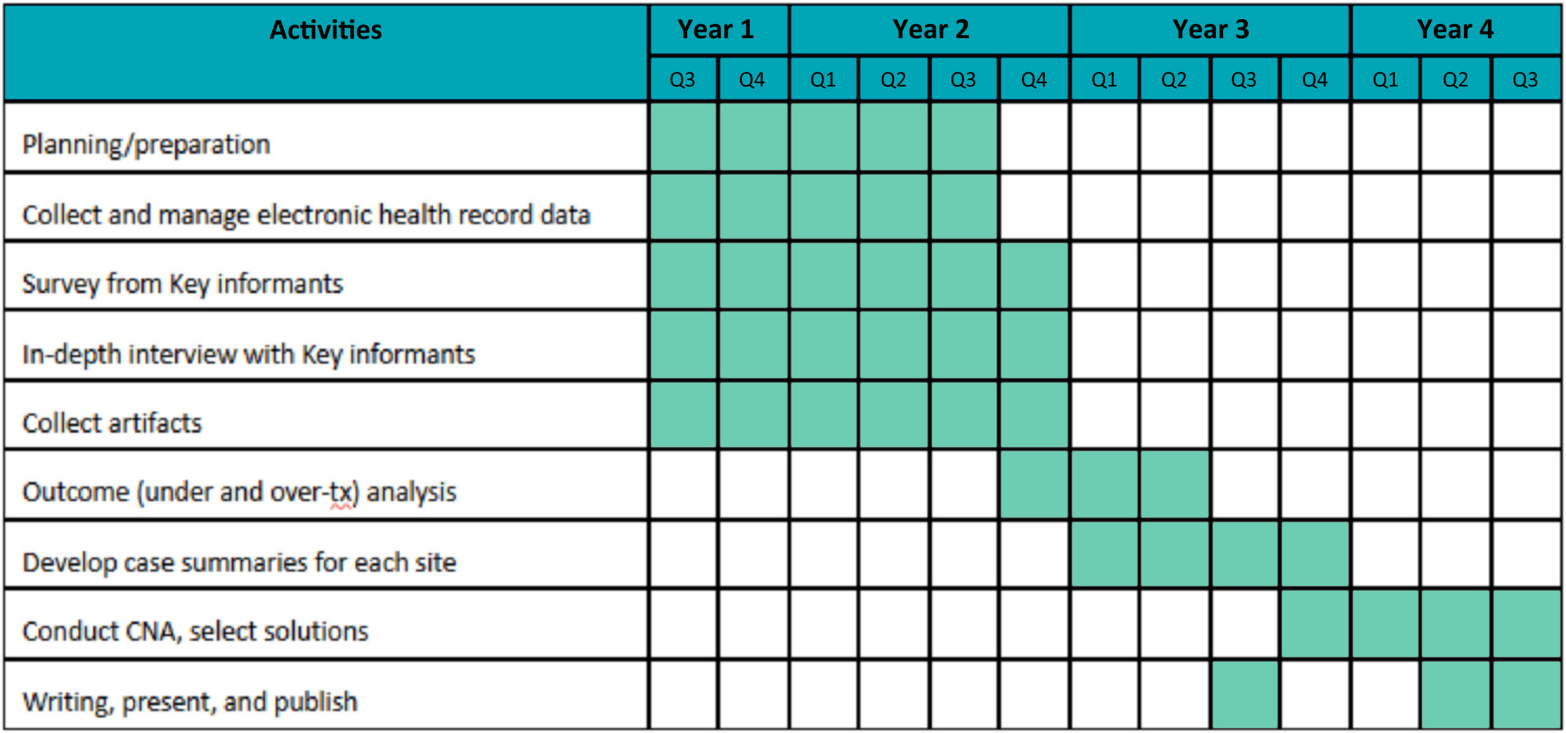
Study timeline.

This study has four data sources: (1) surveys, (2) interviews of ED key informants, (3) artifacts of initiatives intended to improve sepsis diagnostic accuracy (e.g., sepsis committee charters; sepsis coordinator job descriptions) to understand broad factors influencing sepsis diagnostic accuracy, and (4) EHR data to identify hospital‐level variability in outcomes of sepsis undertreatment and overtreatment (see Figure 2: Timeline). We will combine SaferDx^19^ with two implementation theoretical frameworks to understand mechanistic relationships among SaferDx domains (i.e., sociotechnical): The Theoretical Domains Framework (TDF) synthesizes 33 theories of individual behavior and behavior change into 14 domains,^20^ and the Organization Theory for Implementation Science Framework synthesizes nine organization theories into six domains.^21^ The SaferDX is a complex socio‐technical framework that describes the structure, process, and outcomes involved in measuring and improving diagnosis.^19^

#### Surveys

We will identify potential participants for the surveys through our team's deep engagement with system leaders and invite them to participate via email. The survey instruments will include a detailed questionnaire guided by themes from Safer‐Dx^19^ and the TDF.^20^ The survey will be administered through REDCap.^22^ Stakeholders will be reminded to respond to the survey every 2 weeks for up to three contacts. Surveys will be completed with implied consent after adequate information is provided, including the option to decline participation without consequence.

#### Interviews

Based on the initial survey response to identify relevant personnel and our team's knowledge of each hospital's organizational structure, we will identify key informants from each site to conduct individual in‐depth interviews. Interview guides are tailored to the position of the key informant (e.g., system and hospital leaders: external influences on system approaches to improving sepsis diagnostic accuracy; ED leaders: system and hospital influences on ED approaches to improving sepsis diagnostic accuracy). Research staff will contact potential participants via email and will interview those who consent to participate. We will email additional stakeholders identified during the interview to invite them to participate (e.g., snowball sampling).

#### Artifacts

We will ask respondents to provide existing hospital documents, such as hospital infrastructure, practices, and processes relevant to sepsis diagnosis for review.

#### EHR data collection

We will capture granular, routine clinical data from the study hospitals’ EHR and data warehouses to represent sepsis identification and treatment decisions, along with health outcomes, during hospital admission. All study hospitals use EPIC EHR systems. We will utilize the common data structures and concepts in EPIC's relational databases that store relevant clinical data at all study hospitals to standardize data and facilitate mapping to a common format. Below are the variables we will collect for this study:

Patient data: Sociodemographic: Age, sex, race, Area deprivation index Illness severity: Vital signs, lab values, orders.

Medical history: Charlson Comorbidity Index, prior healthcare use.

Hospital data Setting: Bed size, %Medicare, location, rurality affiliation: teaching status.

Complexity: Case‐mix, sepsis case‐volume.

Other data Time: Calendar year, month/season.

Outcomes data Treatment: Antibiotic use, route, timing Clinical: Hospital mortality, intensive care unit transfer, hospital‐free days to day 28.

#### Sample size

The unit of analysis is the hospital in which sepsis diagnostic practices are implemented. Based on historical estimates, we anticipate collecting data from at least 500,000 suspected sepsis hospitalizations at 20 hospitals during the study period. Our large sample size will reduce the level of uncertainty and promote adequate precision of the estimated risk‐adjusted sepsis diagnostic error rates. For the key informants’ survey, we will purposively select 10–15 stakeholders per hospital. Based on prior work, we anticipate a greater than 50% response rate from targeted stakeholders, resulting in at least 100 total responses (5–10 per hospital) and yielding sufficient information from each hospital to create a comprehensive case description with consistent detail for comparison across sites. Using the responses from the initial survey and our team's knowledge of organizational structure at each hospital, we will identify 2–3 individuals from each site to participate in in‐depth interviews. We will continue to recruit interview participants until we reach thematic saturation, that is, the point at which further data collection and analysis identify no new themes.^23^

### Analysis plan

#### Survey

We will aggregate survey responses from key informants within each hospital and report descriptive statistics for each survey item with the hospital as the unit of analysis.

#### Interviews

Key informant interviews will assess Organizational Theory for Implementation Science (OTIS)^21^ constructs to understand inter‐ and intraorganizational factors influencing hospitals’ sepsis diagnostic practices. We will use template analysis^24^ in ATLAS.ti, a software for qualitative analysis. For template analysis, we will develop codes derived from OTIS constructs and will use this template to analyze transcripts to identify key themes related to barriers and facilitators that influence accurate sepsis. We will continue to recruit interview participants until we reach thematic saturation, that is, the point at which further data collection and analysis identify no new themes.

#### Artifacts

We will analyze implementation artifacts using template analysis^24^ in ATLAS.ti (qualitative data management and analysis software), applying codes derived from SaferDx and allowing additional themes to arise during analysis.

#### EHR data

For both co‐primary treatment decision outcomes (i.e., sepsis under‐ and over‐treatment), we will quantify the proportions of patients falling into each error category. To assess the extent of variability in sepsis diagnosis outcomes across hospitals, we will fit two‐level mixed effects logistic regression models to estimate hospital‐specific risk‐standardized sepsis undertreatment and overtreatment rates.^25^ This approach is well‐suited to examining differences between hospitals of varying size, given its ability to account for smaller sample sizes by “shrinking” these estimates toward the overall mean. We will incorporate patient and hospital factors into each model to account for features observed before initial antibiotic administration. We will use the hospital‐specific random intercepts derived from each model to calculate risk‐standardized sepsis under‐ and over‐treatment rates for hospitals (i.e., the hospital‐specific ratio of predicted‐to‐expected errors, multiplied by the unadjusted “error” rate for the overall study population). Using bootstrapping, we will construct 95% confidence intervals for each risk‐standardized estimate to facilitate comparisons across hospitals. Findings will be used to rank hospitals into performance groups based on risk‐standardized sepsis undertreatment and overtreatment rates (i.e., “better than expected”, “as expected”, and “worse than expected”—similar to Center for Medicare and Medicaid Services’ (CMS) hospital comparison strategy).

#### Coincidence analysis

Coincidence analysis is a type of configurational comparative analysis that uses Boolean algebra to identify necessary and sufficient conditions for an outcome. Using a four‐step process (i.e., data integration, outcome calibration, factor selection and calibration, and analysis), we will leverage these analytic strengths to identify specific combinations of conditions that uniquely distinguish hospitals with higher versus lower sepsis diagnostic safety and quality.

#### Data integration (Step 1)

We will integrate the different types of data collected for each study hospital, including (i) survey response, (ii) interview, (iii) artifacts, and (iv) EHR data. We will use the summaries to guide discussions among study investigators during biweekly meetings and to guide cross‐case comparisons with the purpose of describing contextual variation associated with sepsis diagnostic accuracy. We will conduct additional classification and/or transformation of themes to generate dichotomous or categorical scores for each hospital for inclusion as factors in coincidence analysis.

#### Defining the sepsis diagnostic safety and quality (SEP‐DiSQ) outcome (Step 2)

The primary outcome is a combined sepsis diagnostic safety and quality outcome generated by integrating Sepsis Undertreatment and Sepsis Overtreatment (derived from EHR data) into a single metric (SEP‐DiSQ). Because clinicians and policy makers are willing to allow some overtreatment of sepsis to prioritize minimizing undertreatment, the sepsis diagnostic safety and quality outcome is hierarchical. We will first rank hospitals by their risk‐standardized undertreatment rate, then we will rank hospitals by their risk‐standardized overtreatment rate. We will designate “High‐diagnostic accuracy” hospitals as those in the “better than expected” performance group for sepsis undertreatment who are also not in the “worse than expected” performance group for overtreatment. We will designate “Low‐diagnostic accuracy” hospitals as those in the “worse than expected“ performance group for sepsis undertreatment who are also not in the “better than expected” performance group for overtreatment. The remaining hospitals will be designated “Intermediate‐diagnostic accuracy.”

#### Selecting and calibrating conditions to create a data matrix (Step 3)

We will apply standard data reduction strategies for coincidence analysis.^26^, ^27^ We anticipate more than 20 potential explanatory factors will be identified based on theoretical and empirical knowledge of the cases and hypothesized influence on the targeted outcome guided by the SaferDx, TDF, and OTIS conceptual frameworks. An example factor is “accreditation standards are consistent with hospital's approach to sepsis diagnosis and treatment” (yes/no). To reduce the number of factors, we will use the “msc” (minimally sufficient conditions) function in the Coincidence Analysis R package, “cna”,^28^ which allows for simultaneous consideration of all potential explanatory factors across the 20 hospitals to identify distinguishable combinations of conditions that are most strongly linked to the SEP‐DiSQ outcome (i.e., diagnostic accuracy). We will initially apply a consistency threshold criterion for inclusion of 100% and reduce by increments of 5% for subsequent iterations, until one of more configurations satisfies the desired consistency threshold. Under these parameters, we will identify all one‐, two‐, three‐, four‐, and five‐condition configurations (contingent on computational constraints) from the potential explanatory factors that meet the given consistency threshold. Resulting configurations will be rank ordered by coverage (i.e., the proportion of hospitals explained by the solution). We will then review the configuration‐level output to inductively guide selection of the smaller subset of factors for final model development. Nondichotomous conditions will systematically be calibrated using theoretical knowledge and recommended practices to indicate the degree to which the condition is present or absent. Calibrated conditions for each hospital will be compiled into a numerical data matrix along with the coded outcome (high, low, or intermediate diagnostic accuracy) for use in conducting coincidence analysis.

#### Coincidence analyses model development (Step 4)

After factor selection and calibration, we will build final analytic models using the modeling functions in the “cna” package in R. Our approach follows standard procedures as described in ours and others’ prior work, with the goal of identifying causal chains of conditions that are minimally sufficient and necessary to achieve high sepsis diagnostic accuracy. Using this approach, we will select final models based on overall model coverage (e.g., 80% or more), high consistency (e.g., near 100%), and limited model ambiguity.^29^ We will perform sensitivity analyses to assess how well the model performs when the data set excludes patients with septic shock, because there is less treatment uncertainty in patients with shock and the potential harms from undertreatment are much higher relative to overtreatment. Finally, we will compare solution versus nonsolution site differences in downstream outcomes (i.e., hospital mortality, ICU admission, hospital‐free days) using *t* tests.

#### Patient and public involvement

There will be no direct patient or public involvement in this study.

## ETHICS AND DISSEMINATION

The study has been approved by both the University of Michigan (UM) and Wake Forest Institutional Review Boards (IRB). The UM‐IRB board serves as the single‐site IRB for this study. This is a minimal‐risk study that examines routine clinical care without intervention that does not alter the care patients receive. We will obtain consent from interview participants. We will disseminate study findings through our strong existing stakeholder networks that guide practice and policy for sepsis. We will promptly prepare results for presentations at national/international meetings and publications in high‐impact journals to increase the visibility of findings to scientists, evidence consumers, policymakers, and the lay public.

## DISCUSSION

This study aims to understand sepsis diagnostic practices that are context‐specific to achieve high‐performance on both under‐ and overtreatment outcomes. We will ultimately use these findings to develop a toolkit to facilitate the translation of this evidence into practice. Our approach uses tools and principles from safety and implementation science to generate first‐of‐its‐kind evidence to improve diagnostic excellence in sepsis. The study has several strengths: (1) it improves upon existing sepsis diagnosis research by integrating both under‐ and over‐treatment in the evaluation of diagnostic quality, (2) it focuses on sepsis diagnosis as a confluence of individual, team, ED, hospital, and system‐level factors, rather than a solely individual decision‐making process, representing significant advancement over extant context‐agnostic research, (3) it is grounded in multiple complementary conceptual frameworks, SaferDx, TDF and OTIS, and (4) it leverages an innovative causal inference analytic approach that enables identification of multiple solutions representing combinations of sepsis management practices and contextual conditions that could not be derived from traditional methods (e.g., linear regression). Results of the study will be used directly to guide the selection of feasible and important strategies to promote optimal sepsis diagnosis.

## CONFLICT OF INTEREST STATEMENT

Drs. Taylor and Briken report grant support from National Institutes of Health (NIH) outside of the submitted work. Dr. Kowalkowski reports grant support from NIH and Patient‐Centered Outcomes Research Institute outside of the submitted work. Drs. Taylor, Kowalkowski, and Birken received funding from AHRQ. Dr. Palakshappa received funding from NIH outside of the submitted work. Dr. Miller receives research support from Abbott. The remaining authors declare no conflict of interest.

## Supporting information

Supporting information.

Supporting information.

Supporting information.
