# Supplementary material for: Diagnostic safety and quality optimization in sepsis study protocol
Source: J Hosp Med. 2025 Apr 13;20(7):800–7. doi: 10.1002/jhm.70052 (PMC12217410; doi:10.1002/jhm.70052)
Supplement: Supplementary file 1 — Supporting information. [file JHM-20-800-s001.pdf]

Emergency Department Sepsis Practices Survey

The goal of this survey is to understand factors influencing the initiation of a care process for suspected sepsis in emergency departments. The overarching goal of the study is to develop guidance for reducing undertreatment and overtreatment of sepsis presenting to emergency departments. The study team will ensure that your identity and responses are confidential, but here is always a risk of a breach of confidentiality. Your decision to participate will not affect your work, and there will be no penalty for you or your institution, and you will not lose any services, benefits, or rights you would normally have if you choose not to participate in this survey. You may access the complete Institutional Research Board-approved consent document here: (Link to updated IRB approved consent form)As you answer the following questions to the best of your ability, please consider your experience with adults presenting to the emergency department during the last few years. This survey will take about 5-10 minutes.

Please select one response for each of the following questions.

	Excellent	Very good	Good	Fair	Poor	Not applicable
How would you rate your knowledge of the signs and symptoms of sepsis?	○	○	○	○	○	○
How would you rate your skills in identifying potential sepsis?	○	○	○	○	○	○
How would you rate your skills in differentiating sepsis from non-infectious conditions?	○	○	○	○	○	○

Please use this space to elaborate on any of your responses to the previous questions.

____

In your emergency department, is it within a nurse's scope of responsibility to initiate a care process for suspected sepsis? Please select one response.

- ○ Yes
- ○ No
- ○ Unsure

Please indicate whether the following strategies are used in your emergency department. Please select all that apply.

- ☐ Order sets
- ☐ Sepsis detection algorithms
- ☐ Sepsis huddles
- ☐ Sepsis awareness-raising campaigns (e.g., buttons, giveaways, prizes)
- ☐ Nurse-initiated care process for suspected sepsis
- ☐ Code sepsis
- ☐ Sepsis committees
- ☐ Sepsis champions
- ☐ Sepsis coordinator
- ☐ Audit and feedback
- ☐ Incentives (pay bonus, gift card, public recognition) for sepsis-related performance
- ☐ Sepsis-related training and education
- ☐ Pharmacist present in ED
- ☐ Antibiotics stored in ED
- ☐ Other
- ☐ None of the above
- ☐ Unsure

If other, please specify:

____

Please use this space to elaborate on any of your responses to the previous questions.

____

Please select one response for the following question.

	Very often	Often	Sometimes	Rarely	Never	Not applicable
How often do you forget to consider sepsis as a diagnosis?	○	○	○	○	○	○

Please select one response for each of the following questions.

	Very much	Moderately	Somewhat	Very little	Not at all	Not applicable
To what extent do you feel that you are able to initiate a care process for suspected sepsis?	○	○	○	○	○	○
To what extent do you feel that you have the resources (e.g., technology, documents, staff support) necessary to initiate a care process for suspected sepsis?	○	○	○	○	○	○
To what extent do you feel that you have support from your colleagues for initiating a care process for suspected sepsis?	○	○	○	○	○	○
To what extent do you feel that you have support from your superiors for initiating a care process for suspected sepsis?	○	○	○	○	○	○
To what extent do negative emotions (e.g., burnout, stress, fear) prevent you from initiating or care process for suspected sepsis?	○	○	○	○	○	○
To what extent do competing priorities prevent you from initiating a care process for suspected sepsis?	○	○	○	○	○	○

Please select up to three factors that help you in initiating a care process for suspected sepsis.	☐ Potential clinical consequences (e.g., antimicrobial resistance) of initiating a care process for suspected sepsis<br>☐ Potential administrative consequences (e.g., negative impacts on performance measures) of initiating a care process for suspected sepsis<br>☐ Potential clinical consequences (e.g., antimicrobial resistance) of NOT initiating a care process for suspected sepsis<br>☐ Potential administrative consequences (e.g., negative impacts on performance measures) of NOT initiating a care process for suspected sepsis<br>☐ Resources necessary for initiating a care process for suspected sepsis<br>☐ Support from colleagues<br>☐ Support from superiors<br>☐ Emotions (e.g., burnout, stress, fear)<br>☐ Competing priorities<br>☐ Past experiences<br>☐ None of the above<br>☐ Not applicable
Please select up to three factors that prevent you from initiating a care process for suspected sepsis.	☐ Potential clinical consequences (e.g., antimicrobial resistance) of initiating a care process for suspected sepsis<br>☐ Potential administrative consequences (e.g., negative impacts on performance measures) of initiating a care process for suspected sepsis<br>☐ Potential clinical consequences (e.g., antimicrobial resistance) of NOT initiating a care process for suspected sepsis<br>☐ Potential administrative consequences (e.g., negative impacts on performance measures) of NOT initiating a care process for suspected sepsis<br>☐ Resources necessary for initiating a care process for suspected sepsis<br>☐ Support from colleagues<br>☐ Support from superiors<br>☐ Emotions (e.g., burnout, stress, fear)<br>☐ Competing priorities<br>☐ Past experiences<br>☐ None of the above<br>☐ Not applicable

To what extent do you worry about the following consequences of sepsis OVERtreatment? Please select one response for each item.

	Very much	Moderately	Somewhat	Very little	Not at all	Not applicable
Antimicrobial resistance	○	○	○	○	○	○
Fluid overload	○	○	○	○	○	○
Negative impacts on performance measures	○	○	○	○	○	○
Antibiotic adverse events	○	○	○	○	○	○

Unnecessary use of limited resources (e.g. blood culture bottles, fluid shortage)	○	○	○	○	○	○

To what extent do you worry about the following consequences of sepsis UNDERtreatment? Please select one response for each item.

	Very much	Moderately	Somewhat	Very little	Not at all	Not applicable
Adverse patient outcomes (e.g. dialysis, long-term disability, death)	○	○	○	○	○	○
Poor performance on sepsis quality metrics	○	○	○	○	○	○
Financial penalties for missing sepsis	○	○	○	○	○	○
Poor hospital ranking/reputation	○	○	○	○	○	○

Please select one response for each of the following questions.

	Very much	Moderately	Somewhat	Very little	Not at all	Not applicable
To what extent do sepsis-related initiatives interfere with you attending to other clinical initiatives?	○	○	○	○	○	○
To what extent do you feel that you can raise concerns related to how sepsis is managed in your emergency department without fear of negative consequences?	○	○	○	○	○	○
When the emergency department is busy, to what extent do other clinical priorities interfere with the recognition of sepsis signs and symptoms?	○	○	○	○	○	○
To what extent is your emergency department's sepsis care process consistent with the norms of your profession?	○	○	○	○	○	○
To what extent do you feel that you can rely on your colleagues to respond to suspected cases of sepsis?	○	○	○	○	○	○

Please use this space to elaborate on any of your responses to the previous questions.

____

What is the name of the hospital(s) where you currently work?

____

What is your primary position in the emergency department at your hospital(s)? Please select one response.	◯ Physician<br>◯ Advanced Practice Provider<br>◯ Nurse<br>◯ Pharmacist<br>◯ Quality Coordinator<br>◯ Resident<br>◯ Nursing Assistant<br>◯ Paramedic<br>◯ Other
If other, please specify your position:	__________________________________
What is your primary role in the emergency department at your hospital(s)? Please select one response.	◯ Physician Champion<br>◯ Medical Director<br>◯ Associate/Assistant Medical Director<br>◯ Section Head<br>◯ Chief<br>◯ Chair<br>◯ Advanced Practice Provider Lead<br>◯ Nurse Manager<br>◯ Charge Nurse<br>◯ Bedside Nurse<br>◯ Nurse Educator<br>◯ Triage Nurse<br>◯ Quality Leader<br>◯ Bedside Nursing Assistant<br>◯ Bedside ED Paramedic<br>◯ Bedside Physician or APP<br>◯ Other<br>◯ Not applicble
If other, please specify your role:	__________________________________
How long have you been in your role (i.e., how many years since your training)? Please select one response.	◯ Less than 1 year<br>◯ 1-5 years<br>◯ 6-10 years<br>◯ 11-20 years<br>◯ >20 years
How long have you been in your position at this ED? Please select one response.	◯ Less than 1 year<br>◯ 1-5 years<br>◯ 6-10 years<br>◯ 11-20 years<br>◯ >20 years
What is your current gender identity? Please select one response.	◯ Woman<br>◯ Man<br>◯ Non-binary<br>◯ Genderqueer or genderfluid<br>◯ A gender not listed here<br>◯ Prefer not to answer
If a gender not listed here, please specify:	__________________________________

How would you describe yourself? Please select all that apply.	☐ White/Caucasian<br>☐ Black or African American<br>☐ American Indian or Alaskan Native<br>☐ Asian<br>☐ Native Hawaiian or Pacific Islander<br>☐ Other<br>☐ Prefer not to answer
If other, please specify:	__________________________________
Are you Hispanic, Latino, or of Spanish origin? Please select one response.	◯ Yes<br>◯ No<br>◯ Prefer not to answer
Please indicate whether you would consider participating in an interview to help us further understand your hospital's approach to improving sepsis diagnosis.	◯ Yes<br>◯ No<br>(*Note: Expressing willingness to be interviewed does not guarantee you will be picked for the interview.)
Please indicate whether you would like to be entered into a raffle to win one of three $100 gift cards.	◯ Yes<br>◯ No
Please provide your name and email address so a member of the study team can follow up with you.	__________________________________
