# Supplementary material for: Diagnostic safety and quality optimization in sepsis study protocol
Source: J Hosp Med. 2025 Apr 13;20(7):800–7. doi: 10.1002/jhm.70052 (PMC12217410; doi:10.1002/jhm.70052)
Supplement: Supplementary file 2 — Supporting information. [file JHM-20-800-s003.pdf]

Middle Manager (e.g., Regional lead; service line lead; service line director) Interview Guide

Is this still a good time for you? Are you in a place where you can be free from distractions and feel free to give candid responses?

Thank you for agreeing to speak with me today. The purpose of this interview is to help us understand the pressures that you face with respect to improving sepsis diagnostic quality, how you respond to those pressures, and any efforts you make to resist or modify those pressures.

We will be using the information you share with us today to help construct site-based profiles of approaches to improving sepsis diagnosis. While we will be keeping your location tied to your interview, we will remove any personal identifiers so that you will remain confidential in what you share with us. With the information we gain from these profiles, we hope to develop more effective interventions to support high-quality care for patients. Our discussion should last about 30-45 minutes. You may choose to stop the interview at any time, and there is no penalty to you for not completing the interview.

Taking part in this research is not a part of your job duties, and refusing will not affect your job. You will not be offered or receive any special job-related consideration if you take part in this research.
Before we begin, we would like to ask your permission to audio record our discussion. The recording will be used for training and transcription purposes. The audio recordings will be deleted once the project is complete. If you have any questions, I can provide contact information for the **PI [email].**

Do you have any questions before we begin?

Also, I'd like to record this interview for the purposes of analysis. The only people who will have access to the recording or subsequent transcript are study personnel who are trained in maintaining confidentiality in human subject's research. Are you okay with being recorded?

If yes, hit record now.

ANCHOR ON 2021-23 STUDY PERIOD

1. Could you please introduce yourself and your role in your hospital, particularly as it related to sepsis diagnostic quality during the 2021-2023 time period?
2. Thinking about that time period, could you talk about what or who influenced how you, as a [insert role here; e.g., nurse manager] approach sepsis diagnostic quality, if you received any guidance? Include probes:
    a. Top leaders, like CMOs, CNOs?
    b. System-level leaders?
    c. Other ED leaders or managers?
    d. ED providers, staff or administrators?
    e. External organizations (e.g., professional societies)?
    f. Health system priorities and metrics?
3. Via what channels do you get that guidance/pressure/influence? E.g., meetings, committees, publications

4. Now tell me about how you operationalize guidance/pressure/influence in the ED; e.g., incentives; training; public awareness-raising campaigns; pharmacist in ED; antibiotics stored in ED; punishments/negative feedback; sepsis coordinators; other resources; BPAs, audit and feedback; public kudos; sepsis sniffers; see table below. What did you do?

Functions	(baby) functions
**Understand**: Facilitate effective collaboration between individuals involved in the implementation process by building and sustaining trusting relationships and alliances.	● **Raise awareness**: increasing knowledge and understanding.<br>● **Build skills/mastery:** developing competencies and capabilities.<br>● **Develop self/collective efficacy**: achieving a high level of proficiency and confidence in these skills at an individual and/or group level.<br>● **Engage in sensemaking**: engaging in collective interpreting and understanding.<br>● **Document and monitor:** Tracking, observing, or evaluating behaviors, processes, or outcomes.
**Connect:** Promote a deep comprehension of diverse perspectives and co-create a shared sense of problems and solutions.	● **Foster interaction:** encouraging and promoting engagement, communication, and collaboration amongst individuals or groups.<br>● **Cultivate trust:** building and strengthening trust amongst individuals or groups.<br>● **Foster diversity:** encouraging and celebrating diverse perspectives, backgrounds, and experiences.<br>● **Become unified:** connecting people through a common goal or vision to foster a sense of cohesion and collaboration.<br>● **Create a sense of belonging:** fostering and maintaining an environment where people feel included, accepted, valued, respected, and connected to one another.<br>● **Communicate:** Sharing thoughts, feelings, and information.<br>● **Facilitate collaborative governance/decision-making:** defining and supporting collaborative, transparent, respectful, and inclusive deliberation and consensus-building to make shared/collective decisions.<br>● **Cultivate power to/with:** empowering people to collectively make decisions, take action, and work towards common goals related to the initiative.<br>● **Provide emotional support:** Listening and offering empathy, encouragement, and understanding to/with individuals and groups (so people feel heard & valued).

Middle Manager (e.g., Regional lead; service line lead; service line director) Interview Guide

**Inspire**: Inspire, influence, and motivate the implementation team, engage and cultivate implementation champions and leaders, and foster leadership behavior among all individuals involved in implementation.	• **Demonstrate:** showing, modelling, or illustrating something.<br>• **Embody:** Personifying or consistently behaving in ways that are in alignment with the work.<br>• **Normalize (shared/social norms):** demonstrating that ideas and behaviors have become accepted standards.<br>• **Provide social proof/influence:** leveraging people's tendency to follow or copy the actions of others.<br>• **Engage influentially:** creating sparks (e.g., through communicating messages, through connecting with values) that motivate people to action.<br>• **Create a positive environment:** creating a physical or virtual space where people feel valued, safe, supported, respected, included, and motivated to collaborate and do the work.<br>• **Ensure ownership:** protecting and substantiating people's agency and sense of ownership in the work.<br>• **Acknowledge competence and contributions:** Recognizing and celebrating an individual's or group's skills, strengths, contributions, and achievements.
**Enable**: Identify and remove obstacles, leverage resources, and build capacity.	• **Support problem-solving:** providing the tools, resources, and guidance; creating an optimal environment; or identifying, understanding, and resolving issues or challenges effectively.<br>• **Leverage/acquire resources:** obtaining and leveraging funding, tools, materials, human resources, and other necessary supports.<br>• **Advocate (policy outreach, windows of opportunity, etc.):** enacting strategies and actions to promote the importance of people and the work at hand.<br>• **Reinforce:** bolstering or highlighting when behavior changes have occurred.<br>• **Remind:** prompting and reminding.<br>• **Incentivize:** providing rewards and benefits.<br>• **Restrict:** imposing limitations, controls, or boundaries.<br>• **Clarify:** specifying and reducing ambiguity (e.g., providing role clarity).<br>• **Change physical environment:** modifying the physical space, infrastructure, or switching environments.<br>• **Change work structures:** modifying work structure procedures and policies, such as scheduling and staffing structures.
**Transform**: Reflect on their contributions,	• **Shift mindset:** thinking differently about how to approach the work (e.g., through innovation, different attitudes, dealing with uncertainty…).

manage distress, plan for uncertainty, and support people to be agile and adaptive during implementation.	● **Reflexivity:** thoughtfully considering one's own thoughts, experiences, behaviors, and context.<br>● **Intentionality:** taking purposeful actions that align with values and goals.<br>● **Recognize power within:** reflecting on, understanding, and harnessing one's own strengths, abilities, gifts, and potential to approach work more confidently, purposefully, and impactfully.

5. Could you please tell me about anything that makes it difficult to operationalize the guidance that you receive regarding how to approach to sepsis diagnostic quality? E.g., large sepsis volume; boarding; staffing challenges; hardware/EHR issues; discrepancies between actual performance and performance as reported to CMS or other; mode of communication; psychological safety; relationship quality.
    a. What about things that help you operationalize the approach? E.g., multidisciplinary participation in sepsis governance; sepsis-focused governance
6. Now I'm interested in hearing about whether you ever try to provide feedback on, resist, or modify the people or things that influence how you approach sepsis diagnostic quality.
    a. If you do, via what channels do you try to provide feedback on, resist, or modify the people or things that influence how you approach sepsis diagnostic quality? E.g., relationship-building, advocacy, refusal to adhere to guidance, feedback

CLOSING

Those are all of the questions that I had planned for today. Is there anything else that you want to tell me that would help me to understand how you manage and care for patients with sepsis diagnoses? Thank you for your time!
